# The effect of normalization of sodium on bone turnover markers in patients with epilepsy. A randomized single-blinded placebo-controlled trial

**DOI:** 10.1016/j.conctc.2020.100587

**Published:** 2020-06-09

**Authors:** Sarah Seberg Diemar, Niklas Rye Jørgensen, Pia Eiken, Charlotte Suetta, Noémi Becser Andersen, Anne-Sophie Sejling

**Affiliations:** a Department of Neurology, Rigshospitalet Glostrup, Valdemar Hansens Vej 1-23, 2600, Glostrup, Denmark; b OPEN, Open Patient Data Explorative Network, Odense University Hospital/Institute of Clinical Research, University of Southern Denmark, J. B. Winsløws Vej 19, 5000, Odense C, Denmark; c Department of Clinical Biochemistry, Rigshospitalet Glostrup, Valdemar Hansens Vej 1-23, 2600, Glostrup, Denmark; d Department of Endocrinology and Nephrology, Nordsjællands Hospital, Dyrehavevej 29, 3400, Hillerød, Denmark; e Geriatric Research Unit, Department of Internal Medicine, Herlev-Gentofte Hospital, Herlev Ringvej 75, 2720, Herlev, Denmark; f Geriatric Department, Bispebjerg-Frederiksberg Hospital, Nielsine Nielsensvej 7, 2400, Copenhagen, Denmark; g Department of Clinical Physiology, Nuclear Medicine and PET, Rigshospitalet Glostrup, Valdemar Hansens Vej 1-23, 2600, Glostrup, Denmark; h Faculty of Health and Medical Sciences, University of Copenhagen, Blegdamsvej 3B, 2200, Copenhagen, Denmark

**Keywords:** Epilepsy, Metabolic bone disease, Osteoporosis, Hyponatremia

## Abstract

Hyponatremia [p[Na]<136 mmol/L] is an independent risk factor for decreased bone mineral density (BMD). However, whether hyponatremia represents a surrogate marker, or a direct causal relationship to bone loss remains unknown. The aim of the study was to investigate the effect of salt replacement therapy on bone turnover markers (BTM) and BMD in patients with epilepsy and chronic hyponatremia. This prospective single-blinded randomized trial investigated serum BTM and BMD, evaluated by Dual Energy X-ray Absorptiometry (DXA), in 21 patients at baseline and following three months of salt replacement therapy. Patients with two consecutive measurements of hyponatremia prior to baseline and no known osteoporosis were included from the epilepsy out-patient clinic at Rigshospitalet, Denmark. Seven patients were randomized to placebo and 14 to salt intervention. The baseline p[Na] was 134 (130.5–140) mmol/L (median (IQR)). All patients had BTM within age-specific reference ranges at baseline. Following 3 months of intervention with 3–9 g of salt daily there was no difference in levels of procollagen type 1 N-terminal propeptide (P1NP) or C-terminal cross-linking telopeptide of type 1 collagen (CTX) between placebo and intervention. Nor was there any difference in BMD evaluated at the lumbar spine (L_1_-L_4_) or at the femoral neck or total hip. In our study, salt replacement did neither affect BTM nor BMD. However, due to the small size of the study, more studies are needed to further investigate this.

## Highlights

• Patients with epilepsy and hyponatremia have low bone turnover.
• Defining chronic hyponatremia in patients with epilepsy is challenging.
• Bone turnover markers are not affected by salt supplements.

## 1 Introduction

Patients with epilepsy have a greatly increased risk of developing osteoporosis [1]. As many as 80% of patients with epilepsy have decreased bone mineral density (BMD) and more than 30% have actual osteoporosis [2]. The high occurrence appears to be caused by multiple pathophysiological mechanisms where also hyponatremia seems to play a role [3]. Through the past decade accumulating evidence have shown a strong association between hyponatremia and the occurrence of osteoporosis and fractures [[4], [5], [6]]. Several antiepileptic drugs (AEDs), especially enzyme-inducing AEDs (EIAEDs) such as carbamazepine and oxcarbazepine, are known to cause hyponatremia in up to 46% of treated individuals [[7], [8], [9], [10]]. Despite the high occurrence of hyponatremia, the association with osteoporosis in patients with epilepsy has, to our knowledge, not previously been investigated in a prospective clinical trial. Furthermore, the effect of correcting hyponatremia with salt supplements on bone turnover and BMD is unknown. Therefore, the aim of this study was to evaluate the effect on bone turnover of salt replacement therapy in patients with epilepsy and hyponatremia, evaluated by serum bone turnover markers (BTM) cross-linked N-telopeptide of type 1- collagen (CTX) and procollagen type 1 amino-terminal propeptide (P1NP-intact), and bone mineral density (BMD), evaluated by Dual Energy X-ray Absorptiometry (DXA) scan.

## 2 Materials and methods

### 2.1 Inclusion and exclusion criteria

The inclusion criteria were AED treated epilepsy through a minimum of two years, known chronic hyponatremia (p[Na]<136 mmol/L) in two consecutive measurements, age between 18 and 80 years, fluent in Danish and having signed a form of written consent. Patients were excluded if they had known osteoporosis, defined by the WHO definition of site-specific T-score ≤ −2.5 standard deviation (SD) in any of the following sites: lumbar spine L_1_-L_4_, femoral neck and of the total femur. Patients were likewise excluded if they were undergoing treatment for osteoporosis, were known with the diagnosis SIADH, were pregnant or breastfeeding, had severe concomitant disease like cancer or ischemic heart disease, had a known alcohol, drug or medicine abuse or were known with any disease affecting their calcium metabolism (including primary hyperparathyroidism, thyrotoxicosis, myxedema, severe vitamin D deficiency or severe decreased kidney function).

### 2.2 Study design

Patients came fasting (except water and usual medication) to the clinic on the day of randomization. Here a blood sample was taken between 7.30 and 10am to avoid circadian influence on BTM. All samples were measured on Vitros® 5,1 FS/5600 Ortho Clinical Diagnostics, Albertslund, Denmark. Subsequently the patients were asked question on past medical history, both epilepsy- and osteoporosis-related. At the end of the interview BMD was assessed by DXA scan (Lunar Prodigy™ or iDXA™, GE Healthcare, Chicago, USA). Both scanners underwent daily, and weekly calibration and quality control and all scans were performed by trained technicians. Prior to the baseline visit the patients had collected a 24-h urine sample to assess water and salt output.

**Fig. 1:**
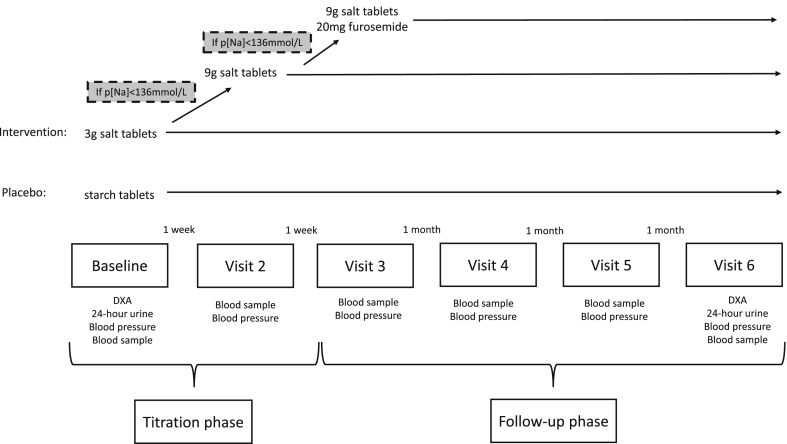
Illustration of the experiment outline. Dual Energy X-ray Absorptiometry (DXA) scan.

Subsequently the patients were randomized into either the placebo or intervention group. Randomization was done by the patient choosing a sealed envelope containing the randomization id. The intervention group was treated with salt tablets (250 mg NaCl pr. tablet (Natriumklorid, MEDA, Allerød, Denmark)), at start dosage 3g/day. Following one week of treatment the patient's p[Na] was measured and if p[Na]<136 mmol/L the salt dosage was increased to 9g/day. Following another week of treatment p[Na] was measured and if p[Na]<136 mmol/L, 1 tablet a day of 20 mg furosemide (Furix, Takeda, Hobro, Denmark) was added to the treatment for the rest of the 3 months follow-up phase. The treatment at which the patients p[Na]≥136 mmol/L was sustained throughout the rest of the study. If the patients p[Na] did not normalize they were sustained throughout the follow-up period on the highest intervention dose of 9g/day of salt and 20 mg/day of furosemide (Fig. 1).

The placebo group was treated with starch tablets containing 51 mg lactose-monohydrat and 120 mg potato starch (Glostrup Apotek, Glostrup, Denmark) throughout the experiment.

At each of the visits a blood sample was taken as described above and at visit 6 the DXA scan and 24-h urine sample was repeated.

### 2.3 Endpoints and statistics

The primary endpoint of the study was the change in bone resorption marker cross-linked N-telopeptide of type 1- collagen (CTX) from baseline visit to visit 6. Secondary endpoints were the bone formation marker procollagen type 1 amino-terminal propeptide (P1NP-intact), and BMD evaluated by Dual Energy X-ray Absorptiometry (DXA).

Due to the exploratory nature of the trial 21 patients were recruited. Patients were randomized 2:1 in favor of the intervention group. A 2:1 inclusion ratio (active vs. control treatment) was chosen in order to increase acceptability for patients to enroll in the study. Since the primary endpoint was relative changes in BTM from baseline to visit 6 men and pre- and postmenopausal women were analyzed together.

To characterize the population all descriptive variables and biochemical parameters were tested for normal distribution and expressed as either mean and standard deviation (SD) or median and interquartile range (IQR) depending on the distribution of data or as N-value and percentage (N (%)). Statistical differences between the placebo and intervention group were for parametric continuous data tested using an independent samples *t*-test and for non-parametric continuous data a Mann-Whitney *U* test was used. For categorical data Pearson chi-square test or Fisher's exact test were used.

To test for any differences in BTM and T-scores between the groups an independent samples *t*-test was done for T-scores and for the BTM a Mann-Whitney *U* test was done. Furthermore, to test for changes from baseline to visit 6 within the two groups, for T-scores a Paired-Samples *t*-test was done and for the BTM a Wilcoxon Signed Ranks test was done.

To further test for any effects of the intervention on BTM or BMD that could not be contributed to the effects of time a factorial repeated measures ANOVA was done.

Data was analyzed using the SPSS software package (version 22.0) (IBM, Armonk, New York). A p-value< 0.05 (two-sided) was considered statistically significant.

### 2.4 Ethics

The study was approved by the Danish Committee on Health Research Ethics (57353) and by the Danish Data Protection Agency (2012-58-0004). Informed consent was obtained from all individual participants included in the study. No experiments and procedures were done that conflict with the Helsinki Declaration of 1975 (revised 2000). The study was registered at clinicaltrials.gov (NCT03371199).

## 3 Results

### 3.1 Baseline characteristics, biochemical parameters, BTM and DXA scans

A total of 21 patients were included in the study. Their osteoporosis-related baseline characteristics and biochemical parameters are shown in Table 1. There was no difference between the groups in age, ethnicity, weight, height, osteoporosis-related characteristics or use of EIAEDs. Nor was there any difference in epilepsy-related characteristics or other biochemical parameters (data not shown). For all patients, the two consecutive measurements of p[Na]<136 mmol/L prior to inclusion were for the first measurement, 132 ± 3 mmol/L (mean ± SD) and for the second 133 ± 3 mmol/L (mean ± SD). Plasma [Na] at baseline was below reference interval for both the placebo p[Na] 133 (130–141) mmol/L (median (IQR)) and intervention group p[Na] 136.5 (130.75–139.5) mmol/L (median (IQR)), with no difference between the groups (p = 0.458). Both the placebo group and the intervention groups had decreased levels of plasma aldosterone with a median of 109 pmol/L and 50.5 pmol/L [ref. interval: 130–859], respectively. Plasma osmolality was decreased for both the placebo, 263.3 ± 30.7 mmol/kg (mean ± SD)), and the intervention group, 277.5 ± 18.1 mmol/kg (mean ± SD)) [ref. interval: 280–300].

**Table 1:** Bone-related characteristics and biochemical parameters at baseline.

Patient characteristics	All patients (N = 21)		Placebo group (N = 7)	Intervention group (N = 14)	Statistical significance Placebo vs Intervention
Female sex (N (%))	16 (76.2)		6 (85.7)	10 (71.4)	0.624
Age at baseline (years) (median (IQR))	48 (42.5–65.5)		48 (35–63)	48.5 (42.75–68.5)	0.799
Ethnicity (N (%))					
Caucasian	20 (95.2)		6 (85.7)	14 (100)	
Asian	1 (4.8)		1 (14.3)	–	0.333
Weight (kg) (mean (SD))	78.3 (15.0)		79.2 (17.8)	77.8 (14.1)	0.839
Height (cm) (mean (SD))	170 (8.4)		166 (5.4)	172 (9.0)	0.129
BMI (kg/m^2^) (mean (SD))	27.0 (4.7)		28.8 (6.9)	26.1 (3.0)	0.352
Family disposition to osteoporosis (N (%))					
No	17 (81)		7 (100)	10 (71.4)	
Yes	4 (19)		0 (0)	4 (28.6)	0.255
Other disease relevant for osteoporosis (N (%))					
No	21 (100)		7 (100)	14 (100)	
Yes	0 (0)		0 (0)	0 (0)	
Has the patient ever received treatment with steroids (N (%))					
No	21 (100)		7 (100)	14 (100)	
Yes	0 (0)		0 (0)	0 (0)	–
History of low energy fractures (N (%))					
No	21 (100)		7 (100)	14 (100)	
Yes	0 (0)		0 (0)	0 (0)	–
History of high energy fractures (N (%))					
No	14 (66.7)		5 (71.4)	9 (64.3)	
Yes	7 (33.3)		2 (28.6)	5 (35.7)	0.572
Intake of calcium (mg/day) (median (IQR))	1169 (804–1521.5)		965 (900–1540)	1210 (676–1543)	0.743
Intake of vitamin D (μg/day) (median (IQR))	29 (5–54)		14 (0–60)	33.5 (14.25–41.00)	0.799
Previous or current smoking (N (%))					
No	10 (47.6)		4 (57.1)	6 (42.9)	
Yes	11 (52.4)		3 (42.9)	8 (57.1)	0.659
Current alcohol consumption (units/week) (median (IQR))					
	4 (0.75–7.5)		4 (0–14)	4 (0.88–7.25)	0.799
Use of EIAEDs (N (%))					
No	2		1	1	
Yes	19		6	13	1.000
**Bone-related biochemical parameters at baseline**					
	Reference interval				
CaI (mmol/L) (mean (SD))	1.18–1.32	1.18 (0.04)	1.19 (0.06)	1.18 (0.03)	0.531
Vitamin D (nmol/L) (median (IQR))	>50	71.0 (49–80)	46.5 (30.75–77.25)	71.0 (56.0–81.5)	0.106
TSH (10^−3^IU/L) (median (IQR))	0.400–4.80	1.92 (1.21–2.33)	2.32 (0.75–2.43)	1.38 (1.22–2.07)	0.322
PTH (pmol/L) (median (IQR))	1.48–7.63	4.30 (3.30–5.38)	4.20 (3.28–5.57)	4.34 (3.21–5.35)	0.856
Phosphate (mmol/L) (median (IQR))	0.76–1.41	1.11 (1.00–1.21)	1.14 (1.09–1.26)	1.09 (0.94–1.21)	0.360
Mg (mmol/L) (mean (SD))	0.71–0.94	0.81 (0.07)	0.81 (0.07)	0.80 (0.08)	0.837
ALP (U/L) (mean (SD))	35–105	60.4 (23.5)	69.1 (27.2)	56.0 (21.2)	0.237
BALP (μg/L) (mean (SD))	8.3–29.4 (F) 7.5–25.1 (M)	15.2 (5.0)	16.9 (6.7)	14.3 (4.0)	0.363

Table 1. Osteoporosis-related descriptive data and blood samples for all patients, the placebo group and the intervention group. All data is number and percentage (N (%)), mean and standard deviation (SD) or median and interquartile range (IQR), when appropriate. Statistical differences between the placebo group and intervention group at baseline were for normally distributed data evaluated by Independent Samples *t*-test, for continuous but not normally distributed variables the Mann-Whitney *U* test was applied. For categorical data the Pearson chi-square test of Fisher's exact test was used, when appropriate. Other diseases relevant for osteoporosis was defined as rheumatoid arthritis, type 1 diabetes, hyperthyroidism, hypogonadism, gastro-intestinal disease, chronic liver disease, pulmonary disease, and organ transplantation. Enzyme-inducing antiepileptic drugs (EIAEDs), Thyroid-stimulating hormone (TSH), parathyroid-stimulating hormone (PTH), ionized calcium (CaI), magnesium (Mg), alkaline phosphatase (ALP), bone specific alkaline phosphatase (BALP).

For levels of ionized calcium (CaI) both groups were in the lower end of the reference range (1.18–1.32 mmol/L), placebo, 1.19 ± 0.06 mmol/L (mean ± SD), and intervention, 1.18 ± 0.03 nmol/L (mean ± SD), with no difference between the groups (p = 0.531). Vitamin D levels (cut-off for sufficient plasma 25-hydroxy vitamin D > 50 nmol/L) in the placebo group, 46.5 (30.75–77.25) nmol/L (median (IQR)), showed hypovitaminosis D as opposed to in the intervention group, 71.0 (56.0–81.5) nmol/L (median (IQR)).

**Fig. 2:**
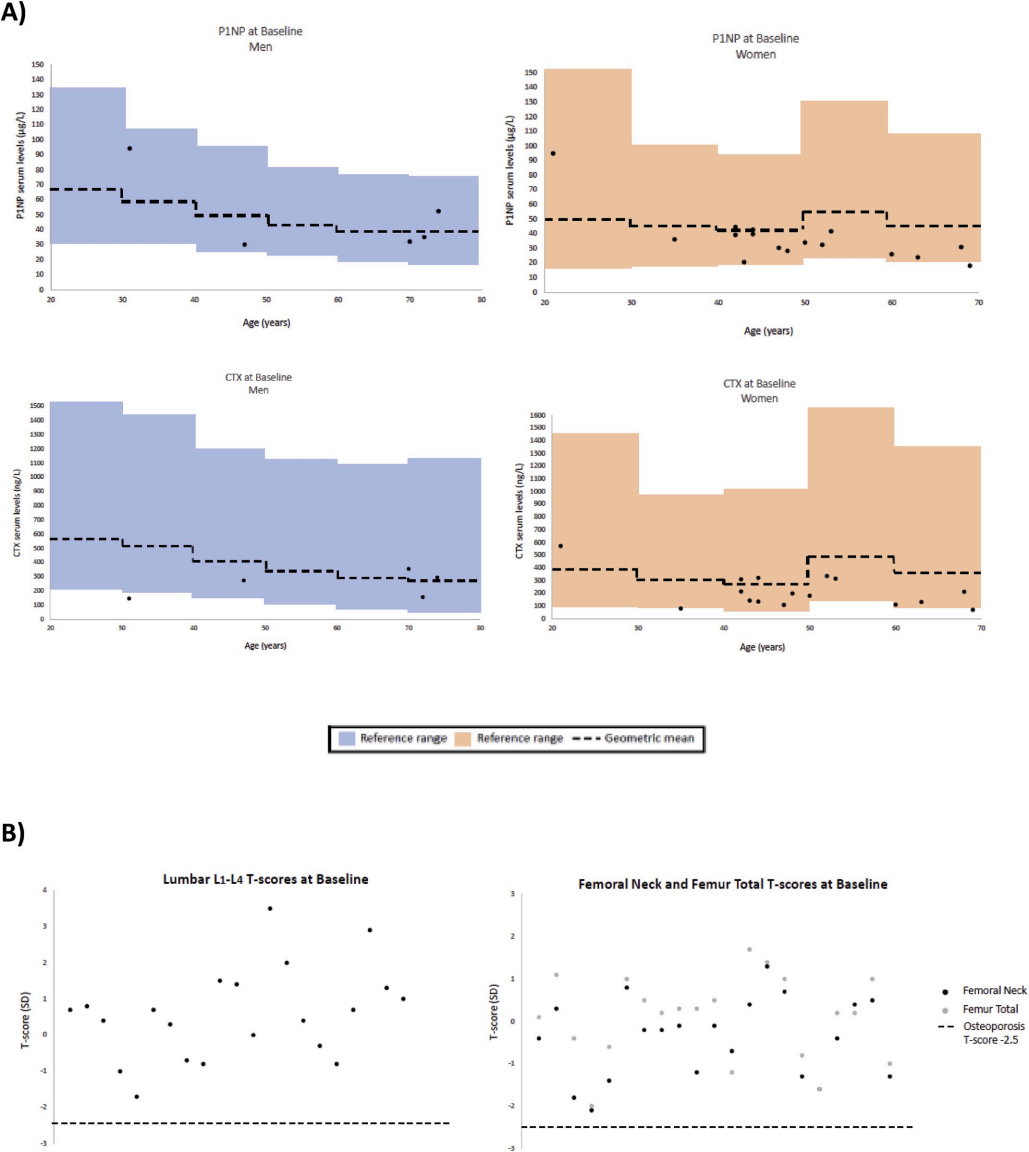
Comparison of bone turnover markers and bone mineral density (BMD) in the study population with the background population. **a:** Procollagen type 1 N-terminal propeptide (P1NP) and C-terminal cross-linking telopeptide of type 1 collagen (CTX) levels in the patients with age-adjusted reference ranges [11]. **b:** Bone mineral density for the lumbar spine L_1_-L_4_ and femoral neck and total femur with cut-off limit for osteoporosis.

All men, across the two groups, except one (20%) with decreased levels of CTX, had baseline plasma concentration of CTX and P1NP within age-specific reference intervals [11]. Two women (13%) had decreased levels of CTX, and one (6%) had decreased levels of P1NP [11] (Fig. 2a).

Due to the inclusion criteria all patients had site-specific T-score > −2.5 SD, with mean T-scores for the lumbar L_1_-L_4_ region, the femoral neck and the total femur of 0.6 ± 0.7 SD (mean ± SD), −0.4±-0.2 SD (mean ± SD) and 0.1 ± 0.2 SD (mean ± SD), respectively (Fig. 2b).

### 3.2 The effects of three months of sodium replacement therapy

**Fig. 3:**
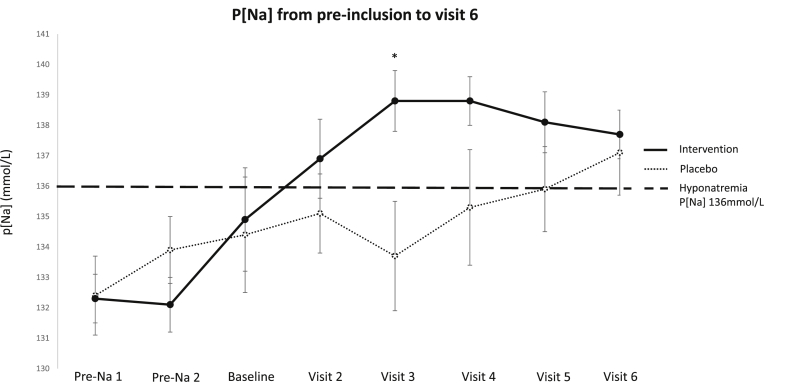
Plasma [Na] for the placebo (dotted line) and intervention group (full line) from pre-inclusion values (Pre-Na 1 and Pre-Na 2) to visit 6. Circles represents mean value and error bars standard error of the mean (SEM). The dashed line represents the definition of hyponatremia (p[Na] 136 mmol/L). The asterix represents a significant difference between intervention and placebo group.

Seven patients were randomized to the placebo group and 14 to the intervention group. Of the 14 patients in the intervention group 7 patients were treated with 3g of salt daily, 3 patients with 9g of salt daily and 4 patients with 9g of salt and 20 mg of furosemide daily. Through the experiment there was only a significant difference in p[Na] at visit 3 between the placebo group 133.7 ± 4.9 mmol/L (mean ± SD) and intervention group 138.8 ± 3.8 (mean ± SD) (p = 0.016) (Fig. 3). Following 3 months of intervention the p[Na] for the placebo group was 137.1 ± 3.7 mmol/L (mean ± SD) and for the intervention group 137.7 ± 2.9 mmol/L (mean ± SD), with no statistical difference (p = 0.706). There was a borderline significant difference in p[Na] following the intervention from baseline to visit 6 for the intervention group (p = 0.050). The compliance in the placebo and intervention group was ≥77% and ≥85%, respectively.

**Fig. 4:**
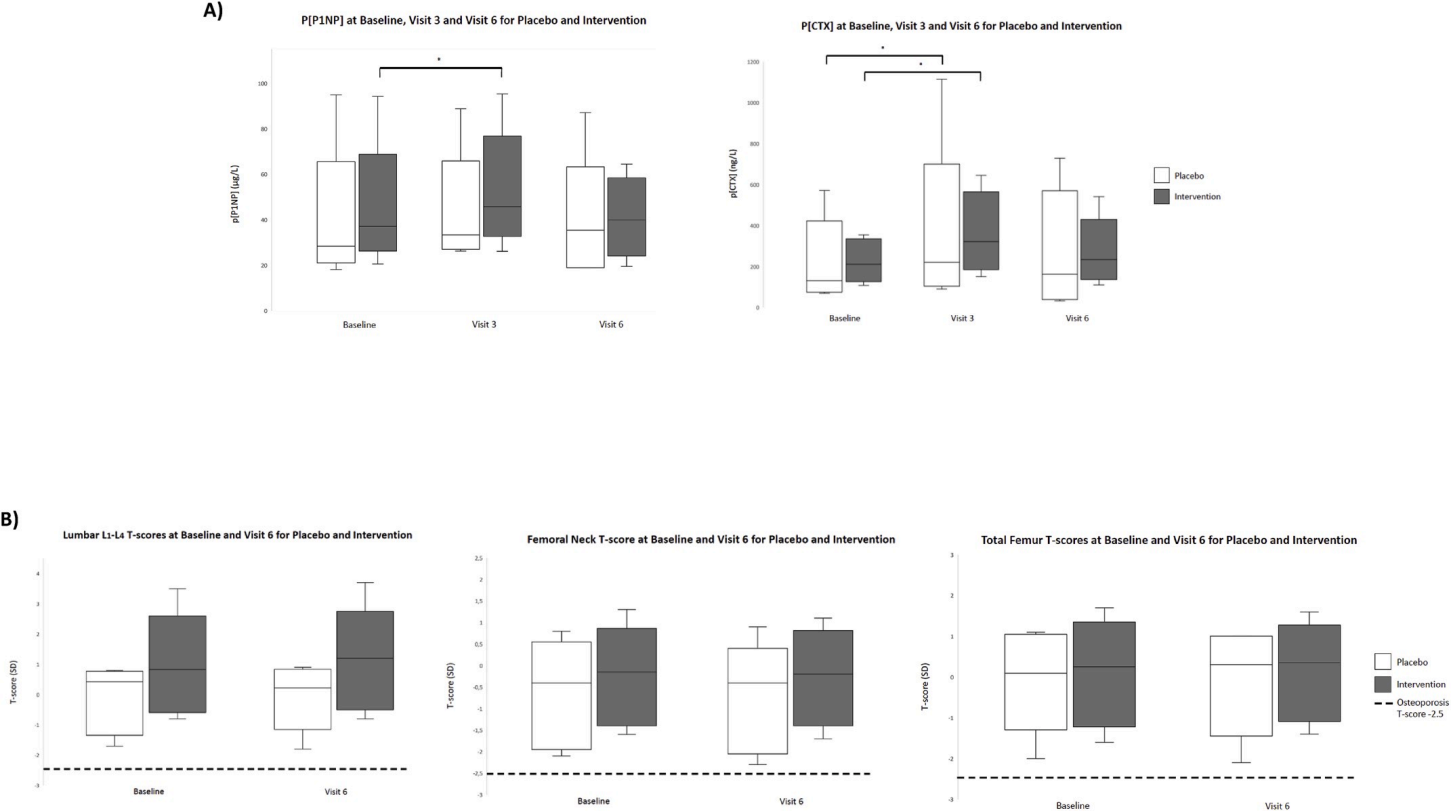
Levels of Procollagen type 1 N-terminal propeptide (P1NP), C-terminal cross-linking telopeptide of type 1 collagen (CTX) and T-scores through the experiment. Boxes represent the interquartile range and the whisker minimum and maximum values, respectively. White boxes represent placebo group and grey boxes the intervention group. **a:** Plasma [P1NP] and [CTX] at baseline, Visit 3 and Visit 6 for the placebo and intervention group. There were significant increases in levels of P1NP and CTX from visit 1 to 3 in the intervention group but only for CTX for the placebo group. There were no other significant differences between the groups. **b:** T-scores at the lumbar L_1_-L_4_ region, the femoral neck and the total femur at baseline and Visit 6. The dashed line represents the cut-off point for the diagnosis of osteoporosis (T-score −2.5). There were no significant differences between the groups.

The results of the BTM and DXA scans are summarized in Table 2. There was no difference between the groups in any of the parameters. Furthermore, there was no difference from baseline to visit 6 in the placebo or the intervention groups in any of the parameters (Fig. 4). When looking specific at visit 3, where a significant difference in p[Na] was present, there is no difference between the placebo and intervention group, but a significant increase in levels of CTX from baseline to visit 3 (p = 0.002) and in P1NP levels (p = 0.001) exists within the intervention group (Fig. 4a). In the placebo groups there was an increase in CTX (p = 0.028) but not in P1NP (p = 0.310) from baseline to visit 3 (Fig. 4a).

**Table 2:** Bone Turnover Markers through the experiment and BMD at baseline and visit 6 for the placebo and intervention group.

	Placebo group	Intervention group	Statistical significance Placebo vs Intervention
**Baseline**			
P1NP (μg/L) (median (IQR))	28.3 (23.9–36.2)	37.1 (31.8–43.3)	0.079
CTX (ng/L) (median (IQR))	131 (80–273)	212 (144.9–315.3)	0.172
L_1_-L_4_ (SD) (mean (SD))	0.0 (1.0)	0.9 (1.4)	0.169
Femoral Neck (SD) (mean (SD))	−0.7 (1.1)	−0.3 (0.9)	0.342
Total Femur (SD) (mean (SD))	0.0 (1.1)	0.2 (1.0)	0.676
**Visit 2**			
P1NP (μg/L) (median (IQR))	35.7 (26.8–46.2)	45.3 (38.0–62.5)	0.149
CTX (ng/L) (median (IQR))	197.1 (125.8–344.1)	369.0 (234.3–469.1)	0.172
**Visit 3**			
P1NP (μg/L) (median (IQR))	33.3 (27.6–43.0)	45.8 (39.2–58.3)	0.056
CTX (ng/L) (median (IQR))	221.2 (117.5–287.0)	321.5 (220.1–485.2)	0.128
**Visit 4**			
P1NP (μg/L) (median (IQR))	34.3 (29.5–40.1)	50.0 (38.8–64.9)	0.110
CTX (ng/L) (median (IQR))	259.2 (142.9–460.5)	331.5 (237.0–440.8)	0.488
**Visit 5**			
P1NP (μg/L) (median (IQR))	37.8 (33.0–48.5)	52.3 (35.4–63.6)	0.287
CTX (ng/L) (median (IQR))	209.5 (130.7–478.1)	344.7 (206.1–476.9)	0.400
**Visit 6**			
P1NP (μg/L) (median (IQR))	35.4 (19–39.4)	39.9 (28.8–52.3)	0.322
CTX (ng/L) (median (IQR))	163 (44.6–411.7)	235.1 (163.3–317.1)	0.488
L_1_-L_4_ (SD) (mean (SD))	0.0 (0.9)	0.9 (1.3)	0.116
Femoral Neck (SD) (mean (SD))	−0.8 (1.2)	−0.2 (0.9)	0.193
Total Femur (SD) (mean (SD))	0.0 (1.1)	0.2 (0.9)	0.615

Table 2. Levels of procollagen type 1 N-terminal propeptide (P1NP) and C-terminal cross-linking telopeptide of type 1 collagen (CTX) expressed as median and interquartile range (IQR). Lumbar L_1_-L_4_ T-score, femoral neck T-score and total femur T-score expressed as mean and standard deviation (SD). Statistical differences between the placebo group and intervention group were for P1NP and CTX tested with a Mann-Whitney *U* test and for Lumbar L_1_-L_4_, femoral neck and total femur an Independent Samples *t*-test was done.

The factorial repeated measures ANOVA shows that, when testing the effect of the intervention on all measures of the same variable over time, there was no effect of the intervention that could not be explained by time, except for femoral neck BMD, where there was an independent effect of the intervention over time (p = 0.040) (data not shown).

For the bone-related biochemical parameters the placebo group had a significantly higher plasma phosphate level, 1.24 ± 0.20 mmol/L (mean ± SD)), than the intervention group, 1.09 ± 0.12 mmol/L (mean ± SD)) (p = 0.044), at visit 6. Levels of TSH increased from baseline to visit 6 in the placebo group, 2.32 (0.75–2.43) 10^−3^IU/L (median (IQR)), to, 3.50 (0.89–4.37) 10^−3^IU/L (median (IQR)) (p = 0.028), while levels of PTH decreased from 4.20 (3.28–5.57) pmol/L (median (IQR)) to 3.60 (2.40–4.25)) pmol/L (median (IQR)), (p = 0.018). For the intervention group there was a significant increase in plasma PTH levels from 4.34 (3.21–5.35) pmol/L (median (IQR)) to 5.60 (3.54–6.22)) pmol/L (median (IQR)) (p = 0.041) and in plasma osmolality from 278 ± 18 mmol/kg (mean ± SD)) to 291 ± 17 mmol/kg (mean ± SD)) (p = 0.027). There was no difference in CaI between the groups at visit 6 nor when comparing within the groups from baseline to visit 6 (data not shown).

Comparing the 24-h urine sample collected at baseline to the one collected at visit 6, the intervention group had a significant increase in U[Na] from 80.9 ± 35.1 mmol/day (mean ± SD)) at baseline to 123.9 ± 40.5 mmol/day (mean ± SD)) at visit 6 (p = 0.001), whereas the placebo groups did not change from baseline 83.1 ± 26.9 mmol/day (mean ± SD)) to visit 6 66.7 ± 26.6 mmol/day (mean ± SD)) (p = 0.159) (data not shown).

## 4 Discussion

To our knowledge this is the first study investigating how hyponatremia affects BTM in patients with epilepsy and whether salt supplements have any effect on BTM in patients with epilepsy and hyponatremia. We did not find elevated levels of the BTM, P1NP and CTX, in patients with epilepsy and chronic hyponatremia and no osteoporosis (Fig. 2a) [11]. Elevated BTM in patients with epilepsy have been found previously however, not in relation to hyponatremia but possibly do to treatment with AEDs [12,13]. There was no change following 3 months of salt replacement in P1NP or CTX levels between the groups nor any changes from baseline to visit 6 (Fig. 4a). We did find a significant difference between baseline and visit 3 in P1NP and CTX levels for the intervention group but only in P1NP levels in the placebo group. There was no difference in T-scores in the lumbar L_1_-L_4_ region, femoral neck or total femur between the groups following the intervention or when comparing within the groups from baseline to visit 6 (Fig. 4b). In the factorial repeated measures ANOVA there was a significant effect of the intervention over time for BMD at the femoral neck.

### 4.1 Hyponatremia-induced osteoporosis in patients with epilepsy

Studies have shown that hyponatremia is associated with fractures and decreases BMD in the general population [[4], [5], [6],^14^,^15^]. Metabolic bone disease has long been known as a grave and frequent co-morbidity to epilepsy and so has chronic hyponatremia, which is associated with certain AEDs [7,16,17]. Not until recently have the two however been combined and the occurrence of moderate and severe hyponatremia [p-Na<129 mmol/L] demonstrated as a risk factor for osteoporosis in patients with epilepsy [3]. Our finding showed no difference in BTM at baseline compared to age-specific reference intervals [11]. However, it should be noted that the patients included in the study did not have osteoporosis, and it is possible that we have selected patients with excellent bone health or a group of “non-progressors” that are less susceptible to adverse effects on bone. The high occurrence of metabolic bone disease in patients with epilepsy had been associated with the use of AEDs and especially those that are EIAEDs [[18], [19], [20], [21]]. These drugs, such as carbamazepine, oxcarbazepine and eslicarbazepine acetate are hypothesized to induce isoenzymes of the livers cytochrome P450 enzyme-system and thereby accelerating hydroxylation of vitamin D and often resulting in hypovitaminosis D in the treated patients [22,23]. This is supported by our finding that the placebo patients had low levels of vitamin D and that the mean level of ionized calcium for all patients was equivalent to the lower end of the reference interval. An interesting overlap exists between the EIAEDs, primarily associated with metabolic bone disease and the AEDs that frequently cause chronic hyponatremia. This complicates the interpretation as it could both mean that hyponatremia plays an independent role in the development of metabolic bone disease or simply serves as a surrogate marker for patients at high risk of developing osteoporosis due to other mechanisms.

A valid question is whether the study population truly represents patients with chronic hyponatremia. The patients were included based on two hyponatremic measurements (Fig. 3), however both the intervention group and the placebo group increased in p[Na] up to baseline indicating that two consecutive measurements of hyponatremia are inadequate to detect patient with chronic hyponatremia and our patients may therefore not be truly chronic hyponatremic. Our patients p[Na] may merely fluctuate around the lower end of the reference interval which is an important point when considering that hyponatremia-induced osteoporosis in patients with epilepsy was demonstrated in patients with moderate and severe hyponatremia (p ≤ 129 mmol/L). The same is found in preclinical studies where the effects in rats is found at p[Na] around 110 mmol and in osteoclasts at media [Na] at 131 mmol/L and more so at media [Na] at 129 mmol/L and lower [5,24]. Furthermore, it is possible that hyponatremia in general does not pose such a great problem in patients with epilepsy as anticipated, which is further illustrated by the fact that we were hardly able to include any patients with moderate or severe hyponatremia.

The question of timely relation is equally important when dealing with hyponatremia-induced osteoporosis. The chronic hyponatremia and the development of osteoporosis are two interlinked chronic conditions evolving over long time periods [6]. It is therefore possible that including patients based on two prior measurements of hyponatremia does not accurately represents the time aspect required for hyponatremia-induced osteoporosis to evolve. This might be a reason why no effects on BTM is detectable.

### 4.2 The effects of salt supplements on BTM and BMD in patients with epilepsy and hyponatremia

The occurrence of hyponatremia-induced osteoporosis has mainly been shown as associations in population-based studies limiting the possibility for drawing conclusions about causality and whether the changes are reversible. There are to our knowledge no studies investigating the reversibility of hyponatremia-induced osteoporosis. In the current study we found no effect on BTM or BMD evaluated by DXA scan following three months of salt supplements. However, when looking at the p[Na] for the two groups through the experiment (Fig. 3) there is a clear increase in the intervention group as well as the placebo group, which spontaneously increase in p[Na] over time. However, the timely difference between the two should be noted, the intervention group increases in p[Na] immediately following the intervention and are normonatremic already at visit 2, whereas the placebo group's increase to normonatremia does not occur until visit 5. This could suggest that the patients included were not truly chronic hyponatremic, however there is no strict definition of chronic hyponatremia in this context. An important point, however, is the increase in urine sodium output in the intervention group from baseline to visit 6, which is not found in the placebo group. This demonstrates that the intervention group did have an increase in salt intake and the placebo group did not.

The unexpected increase in p[Na] in the placebo group limits the possibilities for any general conclusions on the effects of salt supplements in patients with epilepsy and chronic hyponatremia. Furthermore, it should be noted that a time span of 3 months, with great likelihood, is far too short a period to see any changes in BMD evaluated by DXA scan. Evaluating changes in BMD by DXA usually requires time periods of two years or more. Our finding of a difference in BTM between baseline and visit 3 within the intervention and placebo group suggest that BTM might be affected by changes in p[Na] levels, however no generalizable conclusion should be based on this finding.

Our finding of a significant interaction over time on femoral neck BMD in the factorial repeated measures ANOVA is most likely a random finding. The difference is derived mainly by a decrease in the placebo groups BMD and not by an increase in BMD in the intervention group. However, the decrease in BMD cannot be attributed to hyponatremia hence the previously mentioned increase in p[Na] in the placebo groups. Furthermore, it is unlikely that an effect should be seen in femoral neck and not also in total femur just as any changes in BMD in three months is unlikely to be found.

A valid point to consider is the choice of examining effects of hyponatremia by using BTM. To our knowledge no literature exists on how BTM are affected by hyponatremia or how fast any changes would be detectable thus further studies are warranted.

### 4.3 Strengths and limitation

The study's main strength is the thorough characterization of the patients both at baseline and throughout the experiment. The design of the study as a prospective, single-blinded study increases the validity. The patients were included from an out-patients clinic in Denmark allowing for a certain degree of generalizability to other patient populations with epilepsy. Furthermore, generalizability is increased by the fact that only very few patients were excluded on other criteria than not having two consecutive measurements of hyponatremia, suggesting as previously stated that hyponatremia is not that grave a problem as anticipated.

However, there are also certain limitations to the study. The choice of intervention type and dose can be discussed, however, since no effect is observed in the patients other than increased urinary sodium excretion this is probably of little consequence. Despite the attempt to avoid bias in the study the single-blinded design does not eliminate any bias introduced by the investigator. Although the patients were blinded to the intervention the mere fact of being included in a study evaluating BTM and effects of salt supplements might influence their behavior and alter their intake of salt. This is supported by the increase in p[Na] seen in both groups, however since only the intervention group increases in urinary salt output this is likely of no consequence.

## 5 Conclusion

In conclusion, in patients with epilepsy and hyponatremia and no osteoporosis, 3 months of treatment with sodium tablets did not result in significantly higher sodium concentrations compared to the placebo group but instead higher concentration of sodium in the urine. There were also no beneficial effects on BTM or BMD. This suggests, that sodium replacement therapy is insufficient when trying to restore the sodium plasma balance in patients with epilepsy. Future studies are warranted to further understand the meaning of hyponatremia in patients with epilepsy and its consequences for bone loss.

## Funding

The work was founded by 10.13039/100010809Jascha Fonden, Inge Berthelsens Legat from the Danish Epilepsy Association, a grant from the Danish Osteoporosis Association, Grosserer L. F. Foghts Fond, Lennart Grams Mindefond, Frimodt-Heineke Fonden, Kong Christian den Tiendes Fond and Krista and Viggo Petersens Fond.

The sponsors had no role in study design, collection of data, analysis or interpretation of data.

## Declaration of competing interest

Sarah Seberg Diemar has received unrestricted research grants from Eisai Co, Ltd. Anne-Sophie Sejling has since the initiation of the work been employed by Novo Nordisk A/S. Niklas Rye Jørgensen has no conflicts of interest to disclose. Charlotte Suetta has no conflicts of interest to disclose. Pia Eiken is an advisory board member for Amgen Inc. and Eli Lilly A/S, and on the speakers’ bureau for Amgen Inc. and Eli Lilly A/S, and own shares in Novo Nordisk A/S. Noémi Becser Andersen is a lecturer at scientific meetings organized by Eisai Co, Ltd, and has received unrestricted research grants from Eisai Co, Ltd.
